# Risk factors for falls in Parkinson's disease: a cross-sectional observational and Mendelian randomization study

**DOI:** 10.3389/fnagi.2024.1420885

**Published:** 2024-06-10

**Authors:** Yifan Zhang, Yuehui Zhang, Yuexin Yan, Xiangxu Kong, Shengyuan Su

**Affiliations:** ^1^Department of Intensive Care Medicine, ShenzhenBaoan People's Hospital, Shenzhen, China; ^2^Department of Neurological Center, ShenzhenBaoan People's Hospital, Shenzhen, China

**Keywords:** Parkinson's disease, falls, clinical prediction, Mendelian randomization, osteoporosis

## Abstract

**Background:**

Patients with Parkinson's disease (PD) exhibit a heightened risk of falls and related fractures compared to the general population. This study aims to assess the clinical characteristics associated with falls in the patient with PD and to gain further insight into these factors through Mendelian randomization analysis.

**Methods:**

From January 2013 to December 2023, we included 591 patients diagnosed with Parkinson's disease at Shenzhen Baoan People's Hospital. Using univariate and multivariate logistic regression analyses, we identified clinical variables associated with falls. We constructed a nomogram based on these variables and evaluated the predictive efficacy of the model. Additionally, we employed summary statistics from genome-wide association studies to conduct two-sample Mendelian randomization (MR) analyses on key variables influencing falls.

**Results:**

Compared to the control group, we identified osteoporosis, motor dysfunction, higher Hoehn and Yahr scale as significant risk factors for falls in PD patients. Conversely, treatment with levodopa and a higher level of education exhibited a protective effect against the risk of falling. MR analysis further confirmed a causal relationship between osteoporosis, education level and falls in PD patients.

**Conclusion:**

Osteoporosis and educational attainment are correlated with falls in Parkinson's disease.

## Introduction

Parkinson's Disease (PD) is a progressive neurodegenerative disorder predominantly characterized by motor dysfunctions such as tremors, rigidity, bradykinesia and issues with balance and coordination. These symptoms not only diminish the quality of life for patients but also substantially increase the risk of falls. Particularly in PD patients, the incidence of falls is higher compared to age-matched healthy individuals (Bloem et al., 2001), potentially leading to severe consequences such as restrictions in daily activities, heightened fear of falling, increased medical costs. and elevated care needs (Dahodwala et al., 2017; Fasano et al., 2017).

Studies have linked the propensity for falls in PD patients to diminished cholinergic activity, with degeneration of the Pedunculopontine Nucleus (PPN) being a primary cause of impaired postural control and gait dysfunction (Bohnen et al., 2009). Gait freezing and postural abnormalities are strongly associated prognostic factors for falls; gait freezing manifests as difficulty in turning and delayed limb coordination, thereby increasing the risk of falling (Murueta-Goyena et al., 2024). Postural abnormalities are primarily caused by impaired motor autonomy and reactive postural control (Bekkers et al., 2018).

In addition to assessments of balance and gait, current research also encompasses neuropsychological testing, non-motor symptoms, and disease-related variables, with demographic factors often serving as confounding factors in prognostic studies of falls. Although motor dysfunctions may compromise motor control and affect gait, they are frequently omitted in previous prognostic models for falls (Custodio et al., 2016). Furthermore, the predictive role of disease severity in falls remains contentious; the Hoehn and Yahr (H&Y) scale is a significant predictive tool (Kader et al., 2016), while the predictive capability of the Unified Parkinson's Disease Rating Scale (UPDRS) III scale still requires validation (Almeida et al., 2017; Kwon et al., 2021; Lindholm et al., 2021a).

PD patients are prone to fractures when they fall, associated with osteoporosis and reduced bone mass (Invernizzi et al., 2009). In addition to bone density reduction directly caused by PD, other factors such as reduced physical activity, vitamin D deficiency, malnutrition, duration and severity of the disease, old age and low body mass index contribute to the development of osteoporosis in PD patients (Pignolo et al., 2022). As the H&Y stage of PD patients increases, the decline in bone density becomes more pronounced (2023).

This study aims to analyze the clinical characteristics, motor disorder scores, disease stages and demographic data of PD patients to identify potential risk factors associated with falls. Moreover, using Genome-Wide Association Study (GWAS) data for Mendelian randomization (MR) analysis, this study further explores the correlation between relevant variables and falls.

## Materials and methods

### Study design and population

This study is a retrospective cross-sectional analysis of patients diagnosed with PD at Baoan People's Hospital from January 2013 to December 2023. Inclusion criteria were: (1) diagnosed with PD, (2) availability of data. Exclusion criteria included: (1) severe cognitive impairment, (2) history of orthopedic or spinal surgery or other chronic diseases of the musculoskeletal system, (3) history of Deep Brain Stimulation (DBS) surgery, (4) chronic renal failure or cancer, and (5) occurrence of stroke, myocardial infarction, severe liver disease, or cardiac disease within 3 months prior to study enrollment.

### Clinical data collection

Patient data were collected from medical records, including basic demographic information (age, gender, and educational level), medical history (chronic diseases), motor function impairment scores (UPDRS III), H&Y staging, medication usage, laboratory tests, non-motor symptoms (including sleep disorders and anxiety), and personal history (smoking and alcohol consumption). The UPDRS III score was assessed during the medication-off period in patients. Anxiety refers to anxiety disorders and is diagnosed based on psychiatric evaluations using anxiety scales. A diagnosis of anxiety disorder is made when the Hamilton Anxiety Rating Scale (HAMA) score exceeds 7 points. Sleep disorders encompass difficulties in falling asleep and sleep maintenance issues, with the diagnostic criterion being a Pittsburgh Sleep Quality Index score of 8 or higher. Patients were divided into fallers and non-fallers based on whether they had fallen unintentionally onto the ground or another lower surface without overwhelming external force or significant internal events (Emerson, 2023). The study was approved by the Ethics Committee of Baoan People's Hospital in Shenzhen and conducted according to the Declaration of Helsinki standards, with an ethical approval number BYL20240403. This study has been successfully registered with the China Clinical Trial Registry under the registration number ChiCTR2400083288.

### GWAS data sources

For the Mendelian randomization analysis, single nucleotide polymorphisms (SNPs) were used as instrumental variables, combining cross-sectional study results with previous research. Educational attainment and osteoporosis were considered exposure factors, with falls as the outcome variable. Data on educational attainment were derived from the Social Science Genetic Association Consortium (SSGAC), including 766,345 individuals of European descent (Lee et al., 2018). Osteoporosis data were derived from an extensive meta-analysis that identified genetic variants linked to Bone Mineral Density (BMD) at the Femoral Neck (FN), Forearm (FA), and Lumbar Spine (LS) in a cohort of 53,236 individuals of European ancestry (Zheng et al., 2015). Susceptibility to falls was determined using data from a UK Biobank study, which included 461,725 cases of falls.

### Statistical analysis

The cross-sectional data of the included patients were described using frequencies and percentages for categorical data and continuous variables were expressed as mean ± standard deviation. The UPDRS- III and H&Y staging were transformed into dichotomous variables based on the optimal cutoff values corresponding to the maximum Youden Index on the receiver operating characteristic (ROC) curve. Educational level, a multicategory variable, was transformed into dummy variables. The Chi-square test was applied to categorical variables, while the Mann-Whitney U test was used to compare the continuous variables between the fallers and the control group. Univariate regression analysis and LASSO regression were utilized to select variables related to falls, followed by multivariate logistic regression analysis to identify clinical variables associated with falls. The model expressed the magnitude of associations using odds ratios (ORs) and 95% confidence intervals (CIs), with a significance level set at *p* < 0.05. Based on the multivariate analysis, a predictive model was constructed and internally validated using the concordance index (C-index), corrected C-index, calibration curves, clinical impact curve (CIC), decision curve analysis (DCA), and the ROC curve to assess the model's predictive accuracy and consistency. The fit of the model was evaluated with the Hosmer-Lemeshow test to check if the predicted probabilities matched the observed probabilities and a forest plot was created for a visual predictive analysis of the risk factors for falls.

### MR analysis

In this study, MR analysis primarily utilized the Inverse Variance Weighted (IVW) method to assess the causal relationships between genetically predicted osteoporosis, years of education and the risk of falls. Additionally, four complementary MR methods were employed to validate the results of IVW: MR Egger, weighted median, weighted mode, and MR-PRESSO for pleiotropic residuals and outliers. The potential pleiotropic effects in the causal estimates were addressed through sensitivity analyses due to the possibility of bias introduced by pleiotropic instrumental variables in the IVW estimate. Cochran's Q test was used to assess potential heterogeneity. Random-effects IVW analysis adjusted for measured heterogeneity. The MR-Egger intercept was used to estimate the level of pleiotropy among genetic variants (*p* < 0.05) was considered indicative of potential horizontal pleiotropy. MR-PRESSO was also utilized to evaluate the presence of pleiotropy by comparing the observed sum of squared residuals with the expected sum of squared residuals. Additionally, leave-one-out analysis was conducted to determine if the results were driven by individual variants. All statistical analyses were performed using R software version 4.1.2 (R Foundation, Vienna, Austria).

## Results

### Demographic characteristics

**Figure 1:**
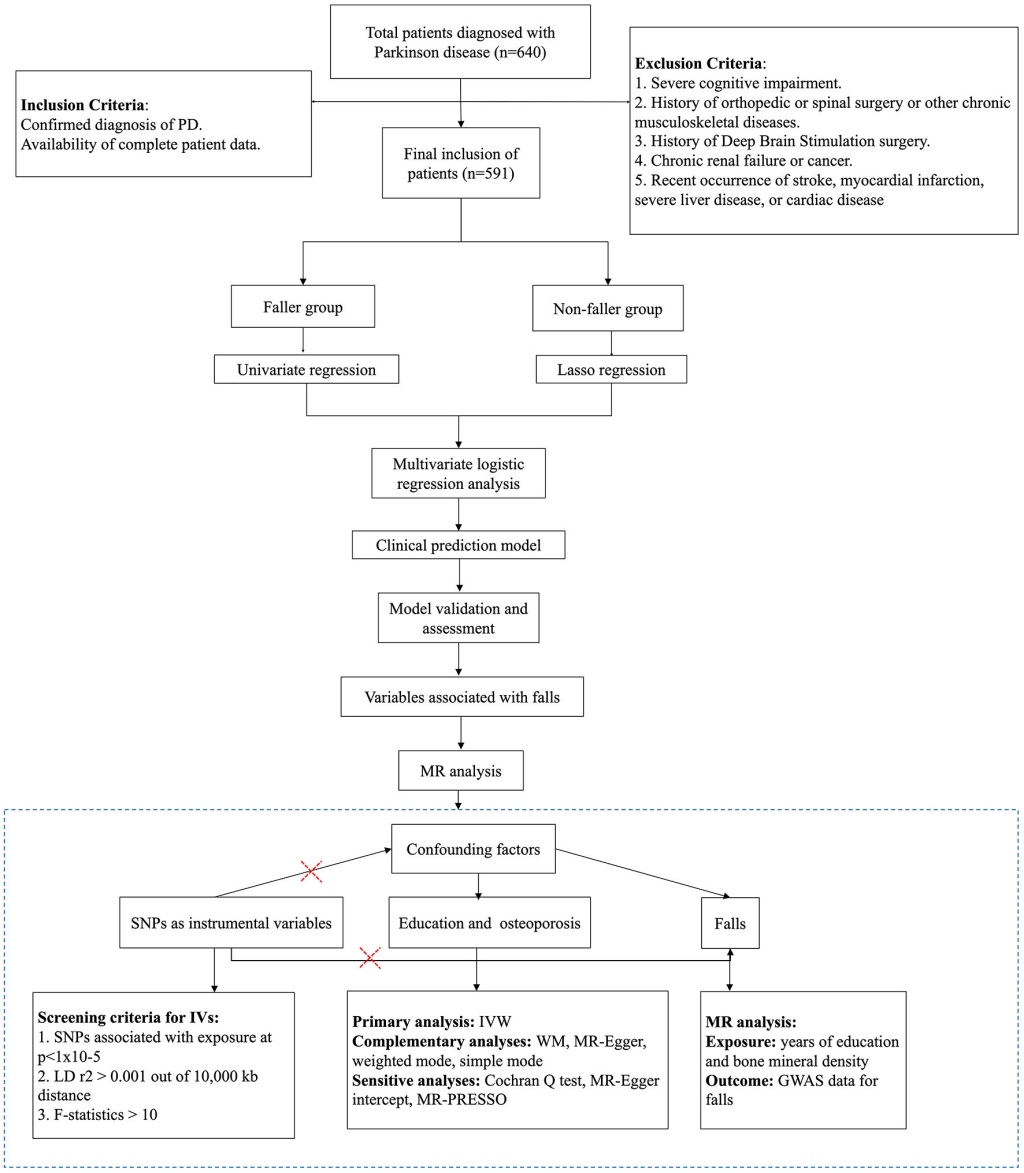
The flowchart of this study, Mendelian randomization (MR).

This study initially collected data on 640 patients. After excluding 8 cases due to DBS surgery, 27 due to joint surgery and 14 due to kidney disease, 591 patients met the inclusion criteria (the selection process is illustrated in Figure 1). These were divided based on the occurrence of falls into a fallers group of 57 patients and a non-fallers group of 534 patients. There were no significant differences in age and gender between the two groups. The educational levels of both groups were predominantly middle to high school, with college-level education or higher present in 21% of the non-fallers and only 5% of the fallers. No significant differences were observed in personal history or laboratory tests between fallers and non-fallers. There was a significantly higher prevalence of osteoporosis in the fallers compared to the non-fallers (faller vs. non-faller: 23% vs. 12%, *p* = 0.027). The proportion of Parkinson's Disease patients with a history of stroke was significantly higher in the fallers than in the non-fallers (faller vs. non-faller: 44% vs. 30%, *p* = 0.039). There was no significant difference in the incidence of hypertension between the two groups. Regarding the severity of motor dysfunction, the fallers had significantly higher UPDRS III scores compared to the non-fallers (faller vs. non-faller: 33 [25, 43] vs. 33 [25, 32], *p* = 0.007). Additionally, the fallers group was at a higher stage of the H&Y scale compared to the non-fallers group (faller vs. non-faller: 2.50 [2.5, 2.7] vs. 2.50 [2, 2.5], *p* < 0.001) (Table 1). In the gender subgroup analysis, males were younger than females, females were more susceptible to osteoporosis compared to males, with a higher intake of calcium supplements (Supplementary Table 1). In the educational level subgroup analysis, individuals with a high school diploma were the youngest, followed by those with a university degree, while those with less than a high school education were the oldest. Within this latter subgroup, the UPDRS III scores were the highest, and there was a greater prevalence of hypertension and coronary heart disease, as well as a higher number of drinkers. Compared to those with more than a high school education, those with less education consumed fewer calcium supplements, yet had lower blood cholesterol levels (Supplementary Table 2).

**Table 1:** Demographic characteristics and clinical features of fallers and non-fallers.

	**Non-fallers (*n* = 534)**	**Fallers (*n* = 57)**	***P* value**
**Demographics**			
Age (years)	77 [69, 84]	78 [69, 84]	0.983
Gender (Male)	301 (56)	27 (47)	0.246
**Motor features**			
MDS-UPDRS III	28 [25, 32]	33 [25, 43]	0.007
Hoehn and Yahr staging	2.50 [2.00, 2.50]	2.50 [2.50, 2.70]	< 0.001
Stage 2	169 (32)	4 (7)	
Stage 2.5	250 (47)	30 (53)	
Stage 3	99 (19)	21 (37)	
Stage 4	16 (3)	2 (4)	
**Education**			0.015
Below high school	265 (50)	31 (54)	
High school	159 (30)	23 (40)	
College or higher	110 (21)	3 (5)	
**Medical history**			
Osteoporosis	62 (12)	13 (23)	0.027
Hypertension	281 (53)	29 (51)	0.911
Diabetes	122 (23)	12 (21)	0.888
CAD	58 (11)	4 (7)	0.501
Stroke	158 (30)	25 (44)	0.039
**Personal history**			
Drinking	15 (3)	4 (7)	0.101
Smoking	11 (2)	2 (4)	0.362
**Sleep and mental health**			
Anxiety	88 (16)	7 (12)	0.528
Sleep disorders	26 (5)	5 (9)	0.208
**Medication usage**			
Levodopa treatment	332 (62)	27 (47)	0.042
**Calcium_Supplement**	38 (7)	3 (5)	0.787
Calcium_Phosphate	38 (7)	3 (5)	0.787
Calcium_Carbonate	150 (28)	22 (39)	0.132
**Laboratory tests**			
WBC ( × 10^9^/L)	4.61 [3.55, 5.77]	6.35 [5.49, 7.63]	0.194
Neutrophils (× 10^9^/L)	4.08 [3.17, 4.79]	4.33 [3.02, 5.48]	0.159
Cholesterol (mmol/L)	0.99 [0.73, 1.3]	3.85 [3.09, 4.59]	0.337
Triglycerides (mmol/L)	2.55 [2.11, 3.08]	1.01 [0.77, 1.28]	0.795
Creatinine (mmol/L)	294.66 [235.05, 366.61]	66 [54.1, 83.1]	0.208
Uricacid (mmol/L)	79.98 [59.25, 84.08]	271.95 [230.4, 366]	0.335

For continuous variable “[ ]”, the values represent quartiles, and for categorical variables “( )” indicate percentages. CAD, Coronary Artery Disease; WBC, White Blood Cell.

### Factors affecting falls in PD

**Figure 2:**
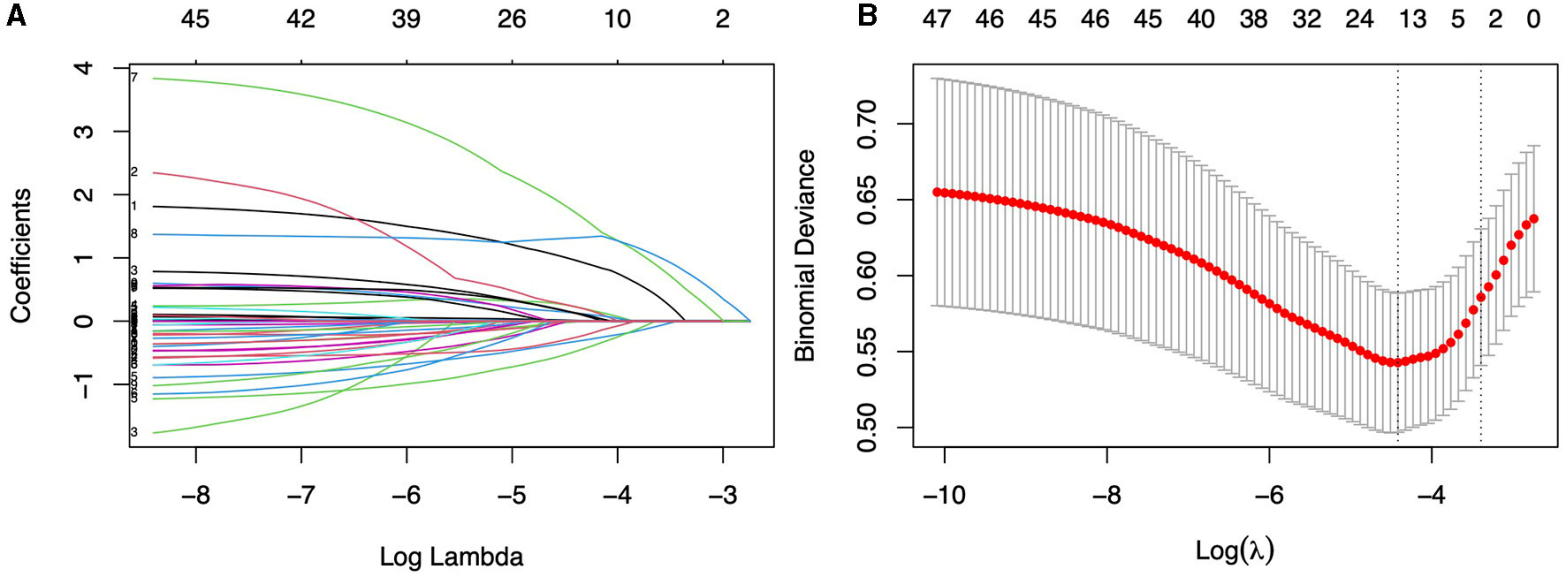
**(A)** Utilizing 10-fold cross-validation to screen for fall-related variables using LASSO regression model. **(B)** LASSO regression cross-validation curve, where the left dashed line represents the lambda value with the best metric evaluation, and the right dashed line represents the lamba value for the model with one standard error of the best value.

**Figure 3:**
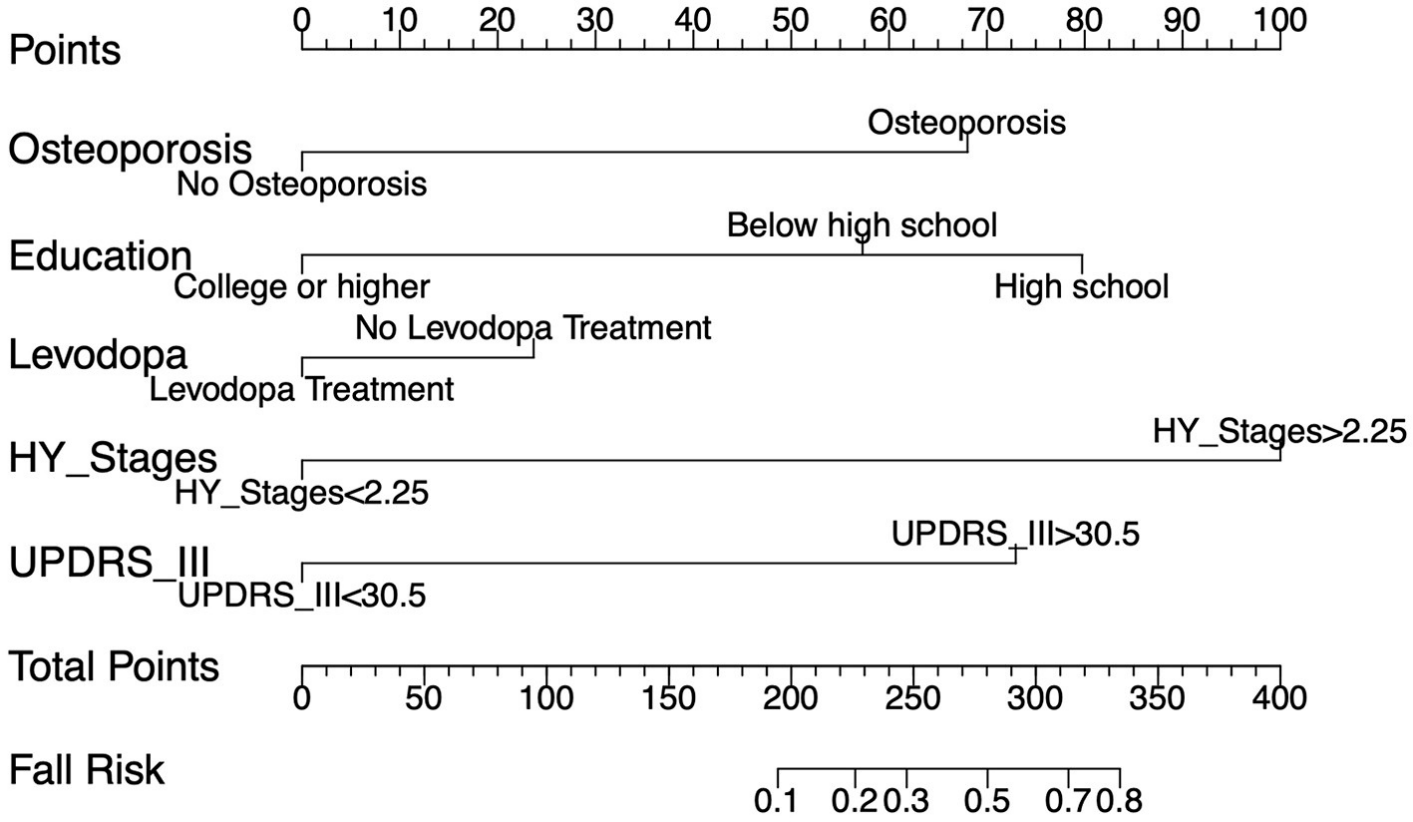
A nomogram illustrating the calculation of risk scores and prediction of fall risk. For each of the five predictive variables, draw a vertical line from the corresponding value to the “Points” line. The sum of these points is then plotted on the “Total Points” line, which correlates to the “Fall Risk” of indicated on the scale.

The ROC curve analysis reveals that the optimal cutoff value for predicting falls based on the UPDRS III score is 30.5, with an area under the curve (AUC) of 0.609 (95% CI, 0.520–0.698). Utilizing this threshold, the sensitivity for diagnosing falls is 59.6%, and the specificity is 74%. The best cutoff value for predicting falls using the H&Y staging is 2.25, with an AUC of 0.657 (95% CI, 0.597–0.717), yielding a sensitivity of 93% and a specificity of 31.6%. A univariate regression analysis was conducted on all demographic variables, clinical motor scores, disease staging, medical history, and medication usage, followed by LASSO regression analysis (Figure 2). Variables with a *p*-value < 0.05 in univariate regression, combined with those selected under the LASSO regression criterion λ-se (λ-se = 0.054), were included in a multivariable logistic regression analysis. Ultimately, the variables associated with falls include levodopa medication use, osteoporosis, educational level, H&Y stages and UPDRS III score. These five variables have been incorporated into the predictive model and depicted in a nomogram (Figure 3). Osteoporosis (OR = 5.56, 95% CI: 2.43–12.77, p = 5.10E-05) significantly increases the risk of falls. Conversely, a higher educational level significantly reduces the risk of falls (OR = 0.25, 95% CI: 0.068–0.90, *p* = 0.034), for individuals with a college education or higher, indicating that higher education acts as a protective factor. Both the H&Y staging (OR = 12.30, 95% CI: 4.14–36.50, *p* = 6.19 × 10^−6^) and UPDRS III scores (OR = 6.401, 95% CI: 3.44–11.94, *p* = 5.07 × 10^−9^) are identified as significant risk factors for falls, with increasing disease progression and motor dysfunction substantially elevating fall risk (Table 2).

**Table 2:** Predictors of falls in univariate and multivariate analysis.

	**Univariate**			**Multivariate**		
	**OR**	**95%CI**	***P*** **value**	**OR**	**95%CI**	***P*** **value**
**Demographics**						
Age (years)	0.967	0.98–1.03	0.82			
Gender (Male)	0.72	0.4–1.2	0.196			
**Motor features**						
UPDRS III (>30.5)	4.201	2.41–7.46	< 0.001	6.407	3.437–11.943	5.07E-09
Hoehn and Yahr staging (>2.25)	6.130	2.46–20.51	< 0.001	12.30	4.142–36.503	6.19E-06
**Education**						
Below high school	Ref.	Ref.	-	Ref.	Ref.	-
High school	1.243	0.69–2.19	0.469	1.760	0.916–3.381	0.090
College or higher	0.234	0.06–0.67	0.018	0.247	0.068–0.900	0.034
**Medical history**						
Osteoporosis	2.250	1.11–4.31	0.018	5.565	2.426–12.767	5.10E-05
Hypertension	0.932	0.54–1.62	0.802			
Diabetes	0.901	0.44–1.7	0.759			
CAD	0.621	0.18–1.58	0.372			
Stroke	1.862	1.06–3.23	0.029	1.580	0.846–2.952	0.152
**Personal history**						
Drinking	2.614	0.72–7.5	0.098			
Smoking	1.727	0.26–6.65	0.484			
**Sleep and mental health**						
Anxiety	0.711	0.29–1.52	0.414			
Sleep_Disorders	1.881	0.62–4.73	0.216			
**Medication usage**						
Levodopa treatment	0.553	0.31–0.95	0.031	0.518	0.279–0.964	0.038
**Calcium_Supplement**	0.728	0.17–2.09	0.602			
Calcium_Carbonate	1.614	0.9–2.81	0.099			

CAD, Coronary Artery Disease; Ref, reference.

### Evaluation of the predictive performance of the nomogram

**Figure 4:**
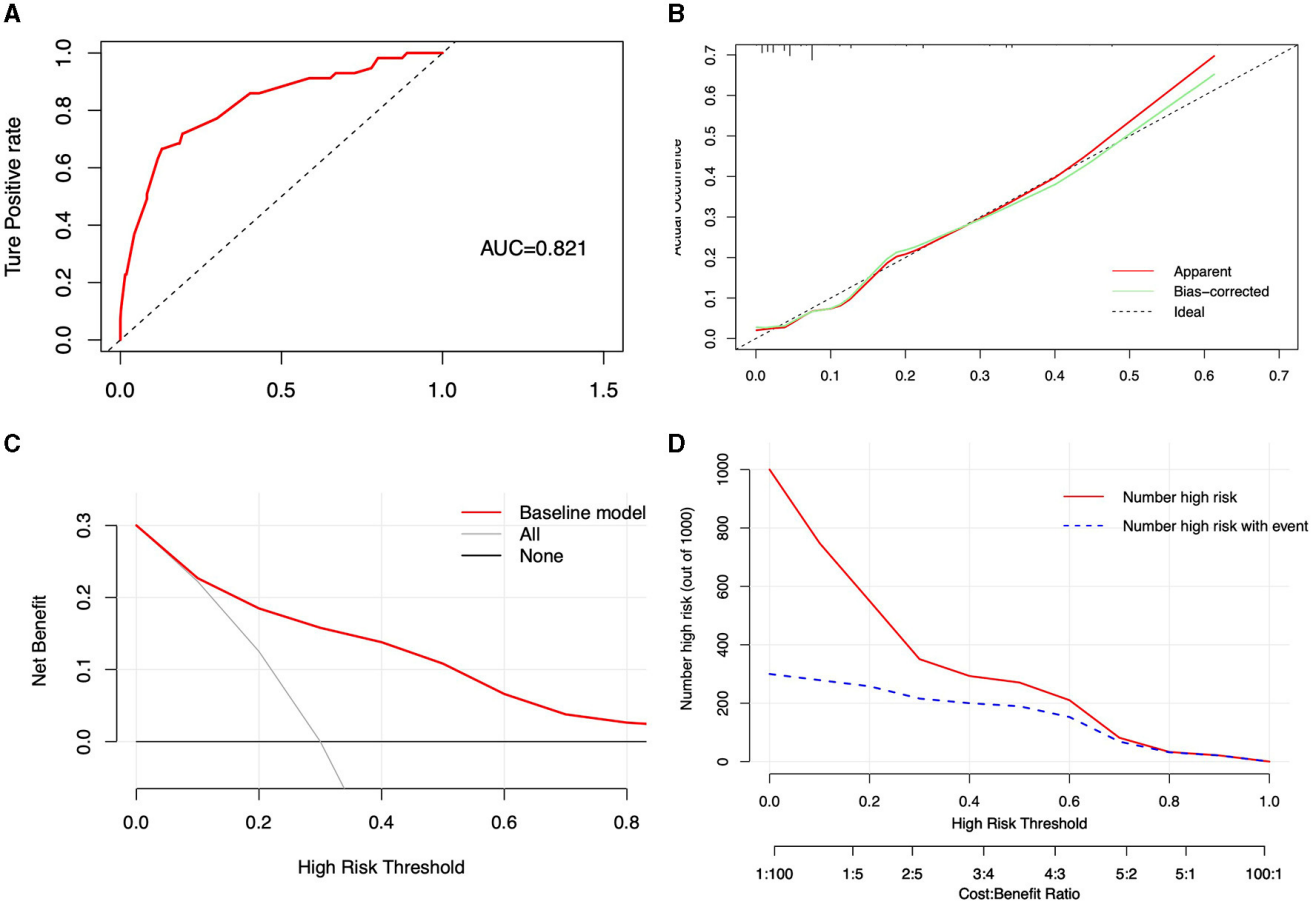
Visualization of the predictive model's performance and clinical impact. **(A)** ROC curve of the predictive model, displaying the area under the curve (AUC); **(B)** Calibration curve for the predictive model, with the current model's performance shown by the red solid line, the adjusted curve by the green solid line, and the ideal line by the black dashed line; **(C)** clinical decision curve of the model; **(D)** clinical impact curve of the model.

The performance of the nomogram model was assessed using the C-index, calculated through the Bootstrap method, resulting in a C-index of 0.821 (95% CI: 0.760, 0.883). Unadjusted and bias-adjusted C-indices were derived using the cross-validation validate method, with values of 0.815 and 0.804, respectively, indicating stable predictive performance in the absence of bias considerations. ROC curve analysis yielded an AUC of 0.821 (Figure 4A), demonstrating the model's excellent discriminative ability. The calibration curve, shown in Figure 4B, along with a Hosmer-Lemeshow goodness-of-fit test *p*-value of 0.078, confirms the model's good calibration. Decision Curve Analysis (DCA) indicates that employing the model (represented by the red solid line) for patient evaluation and intervention at appropriate risk thresholds (>0.1) yields a positive net benefit (NB), compared to scenarios without intervention (black solid line) and complete intervention (gray solid line) (Figure 4C). The Clinical Impact Curve (CIC) at a threshold probability >70% shows a high concordance between the predicted falls and the actual falls, confirming the high clinical efficacy of the predictive model (Figure 4D). Overall, the nomogram provides reasonable and clinically relevant predictions. In the male and female subgroup analyses, UPDRS III scores, H&Y staging, and osteoporosis were identified as risk factors for falls, but a high level of education, calcium supplementation and treatment with levodopa were protective factors only in the male subgroup. Age subgroup analysis showed that in patients with PD both older than 77 years and younger than 77 years, UPDRS III scores, H&Y staging, and osteoporosis remained risk factors for falls. However, treatment with levodopa was a protective factor in patients younger than 77 years (Supplementary Tables 3, 4).

### MR analysis of education, osteoporosis, and falls

Given the significant correlations observed between osteoporosis, years of education and falls in the multivariable regression analysis, we proceeded with MR analysis to infer causal relationships. In the primary IVW analysis, a negative causal relationship was identified between osteoporosis and falls. Specifically, FA-BMD showed an association with falls (OR: 0.996, 95% CI: 0.994–0.998, SE: 0.00091, *p*_adjust < 0.001); LS-BMD (OR: 0.897, 95% CI: 0.978–0.995, SE: 0.004, *p*_adjust = 0.016) and FN-BMD (OR: 0.988, 95% CI: 0.977–0.998, SE: 0.005, *P* = 0.027, *p*_adjust = 0.134). Additionally, years of education demonstrated a significant negative correlation with falls (OR: 0.962, 95% CI: 0.950–0.975, SE: 0.006, *p*_adjust < 0.001) (Table 3). Notably, no evidence of pleiotropy or heterogeneity was detected using the MR-Egger intercept test or the Cochran Q test (both *p* > 0.05). These sensitivity analyses confirm the reliability and stability of the MR results. Supplementary Figure 1 includes funnel plots, leave-one-out SNP analysis and scatter plots to further substantiate these findings.

**Table 3:** Mendelian randomization (MR) analysis of the relationship between years of education, bone mineral density (BMD), and falls.

**Traits**	**Method**	**OR**	**95%CI**	***P* value**	***P* adjust**
Years of schooling	MR Egger	1.000	0.952–1.051	0.997	0.997
Years of schooling	Weighted median	0.961	0.944–0.979	0.000	0.000
Years of schooling	Inverse variance weighted	0.963	0.951–0.975	0.000	0.000
Years of schooling	Simple mode	0.883	0.829–0.940	0.000	0.000
Years of schooling	Weighted mode	0.984	0.931–1.040	0.563	0.703
FA BMD	MR Egger	1.000	0.995–1.004	0.856	0.856
FA BMD	Weighted median	0.998	0.996–1.001	0.165	0.411
FA BMD	Inverse variance weighted	0.996	0.994–0.998	0.000	0.000
FA BMD	Simple mode	0.998	0.992–1.004	0.548	0.684
FA BMD	Weighted mode	0.998	0.993–1.004	0.543	0.684
FN BMD	MR Egger	1.025	0.963–1.090	0.456	0.570
FN BMD	Weighted median	0.989	0.974–1.004	0.150	0.250
FN BMD	Inverse variance weighted	0.988	0.977–0.999	0.027	0.134
FN BMD	Simple mode	0.976	0.950–1.004	0.110	0.250
FN BMD	Weighted mode	1.001	0.972–1.031	0.965	0.965
LS BMD	MR Egger	0.996	0.962–1.031	0.803	0.803
LS BMD	Weighted median	0.991	0.979–1.003	0.152	0.380
LS BMD	Inverse variance weighted	0.987	0.979–0.996	0.003	0.016
LS BMD	Simple mode	0.995	0.972–1.019	0.691	0.803
LS BMD	Weighted mode	0.994	0.974–1.015	0.590	0.803

FA, Forearm; FN, Femoral Neck; LS, Lumbar Spine.

## Discussion

Falls in patients with PD typically impose significant health, economic, and social burdens, particularly leading to decreased independence and reduced quality of life in the elderly (Xu et al., 2022). Patients with PD are more susceptible to injuries and have higher rates of emergency visits due to falls (Dahodwala et al., 2017), making the short-term identification of fall risks crucial for clinical assessments. Long-term, understanding the specific causes of falls in PD patients is vital for predicting and preventing falls, which aids in developing effective fall prevention strategies to mitigate the incidence and related consequences.

In this study, we analyzed clinical data from 591 PD patients to identify factors associated with the risk of falls. The results revealed multiple factors significantly correlated with fall risk in PD patients, including the motor disorder score (UPDRS III), disease staging (H&Y stages), osteoporotic status and years of education. However, given the retrospective nature of our study, causality could not be definitively established. To address this, we integrated a MR analysis using public databases. Although the two-sample MR analysis hinted at potential causal links between years of education, osteoporosis and falls, it is imperative to note that the MR analysis was derived from a European cohort beyond cross-sectional populations. Furthermore, it did not differentiate between Parkinson's and non-Parkinson's cohorts, thus offering a generalized insight into the plausible associations among education level, bone density and fall occurrences. Consequently, the applicability of MR results may be subject to limitations in broader contexts.

Falls and osteoporosis represent major health challenges in PD. More severe motor disorders increase the propensity for falls among PD patients, while osteoporosis heightens the risk of fractures post-fall (Tassorelli et al., 2017). In this study, postmenopausal female patients exhibited a significantly higher incidence of osteoporosis compared to males, with 59% of female patients affected vs. 29% of males. Nevertheless, our study found no gender differences in fall incidents, suggesting the presence of other more influential factors beyond gender. Moreover, the decrease in BMD in PD patients is associated not only with malnutrition and reduced muscle strength but also potentially with long-term levodopa use. Although levodopa is the primary medication improving motor symptoms in PD, it may cause hyperhomocysteinemia, thereby impacting BMD (Lee et al., 2010). Interestingly, despite the potential side effects of levodopa, our study indicates that it also plays a protective role in preventing falls to some extent.

Previous studies have shown that fall frequency is associated with BMD (Fink et al., 2005), a connection further corroborated by our MR analysis. Specifically, the BMD of the ankle and lumbar spine is closely linked to fall events. Consequently, we chose fall incidents occurring within the past year as the study outcome, indicating a possible correlation between recent falls and BMD. Although studies suggest that vitamin D and calcium supplementation can enhance BMD (Voulgaridou et al., 2023), our data revealed no significant differences in calcium supplementation between fallers and non-fallers. Due to the lack of comprehensive BMD data, we could not analyze the relationship between BMD and calcium supplementation in detail. For osteoporosis diagnosis, we relied on the dual-energy X-ray absorptiometry (DXA) measurements of T-scores, with a standard of T-scores lower than −2.5 (Aibar-Almazán et al., 2022). Additionally, we referenced clinical diagnoses by osteoporosis specialists, employing a comprehensive assessment approach to ensure accuracy and thoroughness in diagnoses.

While there is no direct evidence linking educational level with the risk of falls in PD patients, existing studies suggest that a higher educational degree correlates with less severe motor impairments in PD (Kotagal et al., 2015; Jeong et al., 2022). Furthermore, other research indicates that PD patients living in rural areas face a higher risk of falls, likely related to the relatively lower educational levels prevalent in these areas (Xu et al., 2022). MR analysis also supports the protective role of higher educational levels in reducing fall risk. Therefore, future research should further explore the relationship between educational level and fall risk in PD patients.

Studies have shown that the revised H&Y staging is related to the risk of falls in PD patients (Geroin et al., 2020). Although the motor disorder score is considered a crucial risk factor for assessing disease severity, some studies deem it lacks value as a predictive variable (Custodio et al., 2016). Additionally, compared to fall history, cognition, and retropulsion tests, the clinical utility of using UPDRS-III to predict falls appears limited (Lindholm et al., 2021a,b). This study delved into the accuracy of utilizing the UPDRS-III, which is generally reliable but lacks high sensitivity and clear specificity. This implies that while this threshold can adequately identify PD patients who haven't experienced falls, its ability to precisely pinpoint patients prone to falls is moderate. The H&Y staging similarly exhibited limited accuracy in predicting falls. Using 2.25 as the optimal cutoff value notably increased sensitivity but reduced specificity. These findings suggest that although the H&Y staging can identify most at-risk fallers with heightened sensitivity, its low specificity leads to misclassification of many non-fallers as high risk. This could potentially cause undue concern and over-intervention. The AUC analysis mentioned may be influenced by the study's patient count, indicating certain limitations in accurately predicting falls. These findings suggest that multiple factors should be considered in clinical practice to assess fall risk, rather than relying solely on a single assessment tool or disease stage.

This study has certain limitations in assessing non-motor symptoms, particularly the lack of detailed evaluation data on depression and cognitive function. Depression and cognitive impairment are common non-motor symptoms in neurodegenerative diseases like Parkinson's disease, significantly impacting patients' quality of life. Research has shown that higher cognitive reserves are associated with better cognitive function in Parkinson's disease (Gu and Xu, 2022), while significant cognitive deficits also increase the risk of falls in PD (Kim et al., 2013; Cheng et al., 2020). Additionally, PD patients prone to falls often exhibit more depressive symptoms with depression itself being a risk factor for falls in PD (Bryant et al., 2012; Li et al., 2023).

Nonetheless, this study is constrained by its retrospective design and reliance on data from a single regional hospital, leading to potential issues such as selection bias, challenges in establishing causality, and inadequate control of confounding factors. There are challenges such as selection bias, difficulty in establishing causality, and inadequate control of potential confounding factors. Future research should extend to a broader population and multiple centers to validate and deepen these findings. Further studies should also consider including other potential fall risk factors, such as cognitive impairments and visual disorders, BMI, and explore their interactions with the identified risk factors. Additionally, our study did not include patients who underwent orthopedic surgery or deep brain stimulation, lacking analysis for these groups. Overall, this study underscores the importance of comprehensive management of fall risks in PD patients, particularly regarding motor disorders and bone health, helping us better understand and address this increasingly severe public health issue.

## Data availability statement

The original contributions presented in the study are included in the article/Supplementary material, further inquiries can be directed to the corresponding author.

## Ethics statement

The studies involving humans were approved by the Ethics Committee of Shenzhen Baoan People's Hospital. The studies were conducted in accordance with the local legislation and institutional requirements. The participants provided their written informed consent to participate in this study.

## Author contributions

YiZ: Data curation, Investigation, Writing—original draft. YuZ: Methodology, Writing—review & editing. YY: Investigation, Writing—review & editing. XK: Resources, Validation, Writing—original draft. SS: Methodology, Resources, Writing—review & editing.
