# Supplementary material for: Risk factors for falls in Parkinson's disease: a cross-sectional observational and Mendelian randomization study
Source: Front Aging Neurosci. 2024 Jun 10;16:1420885. doi: 10.3389/fnagi.2024.1420885 (PMC11194421; doi:10.3389/fnagi.2024.1420885)
Supplement: Supplementary file 1 [file Table_1.DOCX]

**Supplemental Table1:** Demographic characteristics and clinical features of of male and female.

|  | Female(n = 263) | Male (n = 328) | P value |
| --- | --- | --- | --- |
| **Faller** | 30 (11) | 27 (8) | 0.246 |
| **Demographics** |  |  |  |
| **Age** | 78 (70.5, 85) | 76 (68, 84) | 0.016 |
| **Motor features** |  |  |  |
| MDS-UPDRS III | 28 (24, 33) | 28 (25, 32) | 0.994 |
| Hoehn and Yahr staging |  |  | 0.149 |
| Stage 2 | 84 (32) | 89 (27) |  |
| Stage 2.5 | 111 (42) | 169 (52) |  |
| Stage 3 | 54 (21) | 61 (19) |  |
| Stage 3.5 | 3 (1) | 2 (1) |  |
| Stages 4 | 11 (4) | 7 (2) |  |
| **Education** |  |  | 0.403 |
| Below high school | 137 (52) | 159 (48) |  |
| High school | 82 (31) | 100 (30) |  |
| College or higher | 44 (17) | 69 (21) |  |
| **Medical history** |  |  |  |
| Osteoporosis | 53 (20) | 22 (7) | < 0.001 |
| Hypertension | 148 (56) | 162 (49) | 0.114 |
| Diabetes | 70 (27) | 64 (20) | 0.051 |
| CAD | 31 (12) | 31 (9) | 0.432 |
| Stroke | 67 (25) | 116 (35) | 0.013 |
| **Personal history** |  |  |  |
| Drinking | 0 (0) | 19 (6) | < 0.001 |
| Smoking | 0 (0) | 13 (4) | 0.003 |
| **Sleep and mental health** |  |  |  |
| Anxiety | 52 (20) | 43 (13) | 0.038 |
| Sleep Disorders | 18 (7) | 13 (4) | 0.169 |
| **Medication usage** |  |  |  |
| Levodopa treatment | 153 (58) | 206 (63) | 0.289 |
| **Calcium_Supplement** | 15 (6) | 26 (8) | 0.371 |
| Calcium_Carbonate | 98 (37) | 74 (23) | < 0.001 |
| **Laboratory tests** |  |  |  |
| WBC ($\times$10^9^/L) | 6.35 (5.72, 7.69) | 6.48 (5.64, 7.95) | 0.426 |
| Neutrophils ($\times$10^9^/L) | 4.61 (3.46, 5.52) | 4.61 (3.55, 5.93) | 0.311 |
| Cholesterol (mmol/L) | 4.28 (3.42, 5.01) | 3.86 (3.01, 4.59) | < 0.001 |
| Triglycerides (mmol/L) | 1.01 (0.81, 1.37) | 0.93 (0.69, 1.17) | < 0.001 |
| Creatinine (mmol/L) | 61 (52.26, 72.85) | 77.89 (62, 93.47) | < 0.001 |
| Uricacid (mmol/L) | 277.4 (219.25, 346.8) | 312.2 (247.3, 373.3) | 0.001 |

CAD: Coronary Artery Disease, WBC: White Blood Cell
