# Supplementary material for: Risk factors for falls in Parkinson's disease: a cross-sectional observational and Mendelian randomization study
Source: Front Aging Neurosci. 2024 Jun 10;16:1420885. doi: 10.3389/fnagi.2024.1420885 (PMC11194421; doi:10.3389/fnagi.2024.1420885)
Supplement: Supplementary file 2 [file Table_2.DOCX]

**Supplemental Table2:** Demographic characteristics and clinical features of of patients with different levels of education.

|  | Education degree | | | P value | | |
| --- | --- | --- | --- | --- | --- | --- |
|  | Below high school  (n=296) | High school (n=182) | College or higher (n=113) | Below high school  vs. High school | Below high school  vs. college | High school  vs. college |
| **Faller** | 265 (90) | 23 (13) | 3 (3) | 0.564 | 0.018 | 0.006 |
| **Demographics** |  |  |  |  |  |  |
| Gender (Male) | 82 (45) | 100 (55) | 69 (61) | 0.867 | 0.22 | 0.362 |
| Age | 84 (76, 88) | 67 (63, 78) | 74 (72, 77) | < 0.001 | < 0.001 | <0.001 |
| **Motor features** |  |  |  |  |  |  |
| MDS-UPDRS III | 29 (25, 34) | 27 (24, 31.75) | 28 (25, 30) | 0.002 | 0.205 | 0.137 |
| Hoehn and Yahr staging |  |  |  | 0.495 | 0.372 | 0.324 |
| Stage 2 | 82 (28) | 61 (34) | 30 (27) |  |  |  |
| Stage 2.5 | 140 (47) | 78 (43) | 62 (55) |  |  |  |
| Stage 3 | 63 (21) | 35 (19) | 17 (15) |  |  |  |
| Stage 3.5 | 4 (1) | 1 (1) | 0 (0) |  |  |  |
| Stages 4 | 7 (2) | 7 (4) | 4 (4) |  |  |  |
| **Medical history** |  |  |  |  |  |  |
| Osteoporosis | 43 (15) | 18 (10) | 14 (12) | 0.182 | 0.69 | 0.632 |
| Hypertension | 166 (56) | 83 (46) | 61 (54) | 0.033 | 0.787 | 0.201 |
| Diabetes | 65 (22) | 39 (21) | 30 (27) | 0.982 | 0.394 | 0.385 |
| CAD | 41 (14) | 13 (7) | 8 (7) | 0.036 | 0.086 | 1 |
| Stroke | 99 (33) | 57 (31) | 27 (24) | 0.703 | 0.08 | 0.215 |
| **Personal history** |  |  |  |  |  |  |
| Drinking | 5 (2) | 10 (5) | 4 (4) | 0.041 | 0.269 | 0.627 |
| Smoking | 3 (1) | 6 (3) | 4 (4) | 0.09 | 0.096 | 1 |
| **Sleep and mental health** |  |  |  |  |  |  |
| Anxiety | 46 (16) | 35 (19) | 14 (12) | 0.358 | 0.516 | 0.169 |
| Sleep Disorders | 11 (4) | 14 (8) | 6 (5) | 0.092 | 0.579 | 0.58 |
| **Medication usage** |  |  |  |  |  |  |
| Levodopa treatment | 168 (57) | 121 (66) | 70 (62) | 0.044 | 0.401 | 0.504 |
| **Calcium_Supplement** | 9 (3) | 22 (12) | 10 (9) | < 0.001 | 0.026 | 0.498 |
| Calcium_Phosphate | 9 (3) | 22 (12) | 10 (9) | < 0.001 | 0.026 | 0.498 |
| Calcium_Carbonate | 96 (32) | 45 (25) | 31 (27) | 0.091 | 0.391 | 0.704 |
| **Laboratory tests** |  |  |  |  |  |  |
| WBC ($\times$10^9^/L) | 6.35 (5.76, 8.02) | 6.35 (5.54, 7.76) | 6.4 (5.74, 7.73) | 0.202 | 0.695 | 0.466 |
| Neutrophils ($\times$10^9^/L) | 4.61 (3.53, 5.97) | 4.6 (3.34, 5.38) | 4.45 (3.55, 5.71) | 0.213 | 0.425 | 0.759 |
| Cholesterol (mmol/L) | 3.91 (3.08, 4.61) | 4.21 (3.37, 4.91) | 4.1 (3.37, 5.02) | 0.005 | 0.026 | 0.953 |
| Triglycerides (mmol/L) | 0.97 (0.72, 1.17) | 1.01 (0.7, 1.34) | 1.01 (0.79, 1.39) | 0.225 | 0.046 | 0.401 |
| Creatinine (mmol/L) | 69 (55.38, 88) | 68.15 (57.07, 85.57) | 68 (57, 84.01) | 0.788 | 0.909 | 0.921 |
| Uricacid (mmol/L) | 284.45 (224.9, 362.88) | 293.55 (235.1, 372.48) | 308.1 (247.4, 367.9) | 0.51 | 0.134 | 0.377 |

CAD: Coronary Artery Disease, WBC: White Blood Cell
