# Supplementary material for: Risk factors for falls in Parkinson's disease: a cross-sectional observational and Mendelian randomization study
Source: Front Aging Neurosci. 2024 Jun 10;16:1420885. doi: 10.3389/fnagi.2024.1420885 (PMC11194421; doi:10.3389/fnagi.2024.1420885)
Supplement: Supplementary file 3 [file Table_3.docx]

**Supplemental Table 3:** Impact of various variables on fall risk in gender subgroups of Parkinson's Disease

|  | Male | | | | | | |  | Female | | | | | | |
| --- | --- | --- | --- | --- | --- | --- | --- | --- | --- | --- | --- | --- | --- | --- | --- |
|  | Univariate | | |  | Multivariate | | |  | Univariate | | |  | Multivariate | | |
|  | OR | 95%CI | P value |  | OR | 95%CI | P value |  | OR | 95%CI | P value |  | OR | 95%CI | P value |
| **Demographics** |  |  |  |  |  |  |  |  |  |  |  |  |  |  |  |
| Age (years) | 1.02 | 0.99-1.06 | 0.206 |  |  |  |  |  | 0.98 | 0.95-1.01 | 0.23 |  |  |  |  |
| **Motor features** |  |  |  |  |  |  |  |  |  |  |  |  |  |  |  |
| UPDRS III (>30.5) | 8.14 | 3.52-20.49 | <0.001 |  | 11.287 | 4.428-28.772 | 0.000 |  | 2.28 | 1.05-4.95 | 0.035 |  | 3.552 | 1.485-8.499 | 0.004 |
| Hoehn and Yahr staging (>2.25) | 3.2 | 1.08-13.71 | 0.063 |  | 6.675 | 1.723-25.861 | 0.006 |  | 16.05 | 3.34-288.48 | 0.007 |  | 31.997 | 4.036-25.688 | 0.001 |
| **Education** |  |  |  |  |  |  |  |  |  |  |  |  |  |  |  |
| Below high school | Ref. | Ref. | - |  | Ref. | Ref. | - |  | Ref. | Ref. | - |  | Ref. | Ref. | - |
| High school | 1.64 | 0.71-3.64 | 0.231 |  | 1.760 | 0.916-3.381 | 0.090 |  | 1.55 | 0.69-3.37 | 0.271 |  | 2.345 | 0.916-5.999 | 0.075 |
| College or higher | 0.36 | 0.09-0.8 | 0.049 |  | 0.497 | 0.261-0.949 | 0.034 |  | 0.57 | 0.23-1.07 | 0.135 |  | 0.647 | 0.278-1.507 | 0.313 |
| **Medical history** |  |  |  |  |  |  |  |  |  |  |  |  |  |  |  |
| Osteoporosis | 1.12 | 0.17-4.16 | 0.879 |  | 5.565 | 2.425-12.767 | 0.000 |  | 0.214 | 1.14-5.87 | 0.02 |  | 6.415 | 2.346-17.543 | <0.001 |
| Hypertension | 0.95 | 0.43-2.09 | 0.893 |  |  |  |  |  | 0.87 | 0.41-1.9 | 0.73 |  |  |  |  |
| Diabetes | 0.93 | 0.3-2.39 | 0.892 |  |  |  |  |  | 0.82 | 0.31-1.92 | 0.666 |  |  |  |  |
| CAD | 0 | 0 | 0.989 |  |  |  |  |  | 1.17 | 0.33-3.31 | 0.78 |  |  |  |  |
| Stroke | 1.79 | 0.8-3.96 | 0.151 |  | 1.580 | 0.846-2.952 | 0.152 |  | 2.16 | 0.96-4.72 | 0.057 |  | 1.941 | 0.788-4.782 | 0.149 |
| **Personal history** |  |  |  |  |  |  |  |  |  |  |  |  |  |  |  |
| Drinking | 3.32 | 0.89-10.05 | 0.047 |  |  |  |  |  | - | - | - |  |  |  |  |
| Smoking | 2.11 | 0.32-8.43 | 0.349 |  |  |  |  |  | - | - | - |  |  |  |  |
| **Sleep and mental health** |  |  |  |  |  |  |  |  |  |  |  |  |  |  |  |
| Anxiety | 0.51 | 0.08-1.79 | 0.368 |  |  |  |  |  | 0.79 | 0.26-2.02 | 0.651 |  |  |  |  |
| Sleep_Disorders | 2.11 | 0.32-8.43 | 0.349 |  |  |  |  |  | 1.61 | 0.36-5.29 | 0.471 |  |  |  |  |
| **Medication usage** |  |  |  |  |  |  |  |  |  |  |  |  |  |  |  |
| Levodopa treatment | 0.52 | 0.23-1.15 | 0.105 |  | 0.518 | 0.279-0.964 | 0.038 |  | 0.17497747 | 0.27-1.27 | 0.178 |  | 0.657 | 0.277-1.559 | 0.341 |
| **Calcium_Supplement** | 0.42 | 0.02-2.14 | 0.41 |  |  |  |  |  | 1.21 | 0.18-4.68 | 0.809 |  |  |  |  |
| Calcium_Carbonate | 1.22 | 0.46-2.89 | 0.663 |  |  |  |  |  | 1.81 | 0.84-3.91 | 0.129 |  |  |  |  |
