# Supplementary material for: Risk factors for falls in Parkinson's disease: a cross-sectional observational and Mendelian randomization study
Source: Front Aging Neurosci. 2024 Jun 10;16:1420885. doi: 10.3389/fnagi.2024.1420885 (PMC11194421; doi:10.3389/fnagi.2024.1420885)
Supplement: Supplementary file 4 [file Table_4.docx]

**Supplementary Table 4:** Analysis of the effects of covariates on fall risk in age subgroups of Parkinson's Disease

| Age (years) | $\geq$77 | | | | | | | | <77 | | | | | | | |
| --- | --- | --- | --- | --- | --- | --- | --- | --- | --- | --- | --- | --- | --- | --- | --- | --- |
|  | Univariate | | |  | Multivariate | | | |  | Univariate | | |  | Multivariate | | |
|  | OR | 95%CI | P value |  | OR | 95%CI | P value | |  | OR | 95%CI | P value |  | OR | 95%CI | P value |
| **Demographics** |  |  |  |  |  |  |  |  | |  |  |  |  |  |  |  |
| Gender (male) | 1.1 | 0.51-2.37 | 0.807 |  |  |  |  |  | | 0.38 | 0.16-0.85 | 0.02 |  |  |  |  |
| **Motor features** |  |  |  |  |  |  |  |  | |  |  |  |  |  |  |  |
| UPDRS III (>30.5) | 5.82 | 2.62-13.93 | <0.001 |  | 10.919 | 4.403-27.073 | 0.000 |  | | 2.86 | 1.24-6.54 | 0.012 |  | 4.623 | 1.772-12.065 | 0.002 |
| Hoehn and Yahr staging (>2.25) | 1.3 | 2.71-23.97 | 0.012 |  | 35.997 | 4.551-28.739 | 0.001 |  | | 3.88 | 1.3-16.67 | 0.031 |  | 8.471 | 1.824-39.334 | 0.006 |
| **Education** |  |  |  |  |  |  |  |  | |  |  |  |  |  |  |  |
| Below high school | Ref. | Ref. | - |  | Ref. | Ref. | - |  | | Ref. | Ref. | - |  | Ref. | Ref. | - |
| High school | 1.59 | 0.6-3.78 | 0.319 |  | 1.398 | 0.472 | 0.545 |  | |  | 1.02-5.44 | 0.049 |  | 2.906 | 0.915-9.232 | 0.071 |
| College or higher | 0 | 0 | 0.989 |  | 0.000 | 0.000 | 0.986 |  | |  | 0.27-0.95 | 0.058 |  | 0.876 | 0.405-1.896 | 0.738 |
| **Medical history** |  |  |  |  |  |  |  |  | |  |  |  |  |  |  |  |
| Osteoporosis | 1.64 | 0.62-3.9 | 0.29 |  | 3.932 | 1.230-12.566 | 0.021 |  | | 1.18 | 0.51-2.66 | 0.688 |  | 21.725 | 4.716-100.088 | 0.000 |
| Hypertension | 0.64 | 0.3-1.41 | 0.259 |  |  |  |  |  | | 1.88 | 0.7-4.57 | 0.183 |  |  |  |  |
| Diabetes | 0.49 | 0.16-1.23 | 0.16 |  |  |  |  |  | | 1.86 | 0.28-7.45 | 0.438 |  |  |  |  |
| CAD | 0.33 | 0.05-1.17 | 0.143 |  |  |  |  |  | | 1.17 | 0.33-3.31 | 0.78 |  |  |  |  |
| Stroke | 1.97 | 0.91-4.24 | 0.082 |  | 1.443 | 0.601-3.463 | 0.412 |  | | 2 | 0.84-4.58 | 0.107 |  | 1.740 | 0.667-4.539 | 0.258 |
| **Personal history** |  |  |  |  |  |  |  |  | |  |  |  |  |  |  |  |
| Drinking | 3.57 | 0.5-17.44 | 0.139 |  |  |  |  |  | | 2.29 | 0.34-9.53 | 0.307 |  |  |  |  |
| Smoking | 8.76 | 0.34-225.48 | 0.129 |  |  |  |  |  | | 1.1 | 0.06-6.2 | 0.931 |  |  |  |  |
| **Sleep and mental health** |  |  |  |  |  |  |  |  | |  |  |  |  |  |  |  |
| Anxiety | 0.65 | 0.15-1.98 | 0.504 |  |  |  |  |  | | 0.81 | 0.23-2.23 | 0.708 |  |  |  |  |
| Sleep_Disorders | 2.53 | 0.37-11.09 | 0.261 |  |  |  |  |  | | 2.1 | 0.46-6.95 | 0.269 |  |  |  |  |
| **Medication usage** |  |  |  |  |  |  |  |  | |  |  |  |  |  |  |  |
| Levodopa treatment | 0.9 | 0.42-1.97 | 0.788 |  | 0.604 | 0.245-1.488 | 0.273 |  | | 0.36 | 0.15-0.81 | 0.015 |  | 0.379 | 0.150-0.959 | 0.041 |
| **Calcium_Supplement** | 2.9 | 0.14-23.47 | 0.364 |  |  |  |  |  | | 0.56 | 0.09-2.02 | 0.449 |  |  |  |  |
| Calcium_Carbonate | 1.45 | 0.66-3.11 | 0.341 |  |  |  |  |  | | 1.89 | 0.77-4.39 | 0.147 |  |  |  |  |
