# Supplementary material for: Risk factors for falls in Parkinson's disease: a cross-sectional observational and Mendelian randomization study
Source: Front Aging Neurosci. 2024 Jun 10;16:1420885. doi: 10.3389/fnagi.2024.1420885 (PMC11194421; doi:10.3389/fnagi.2024.1420885)
Supplement: Supplementary file 5 [file Data_Sheet_1.PDF]

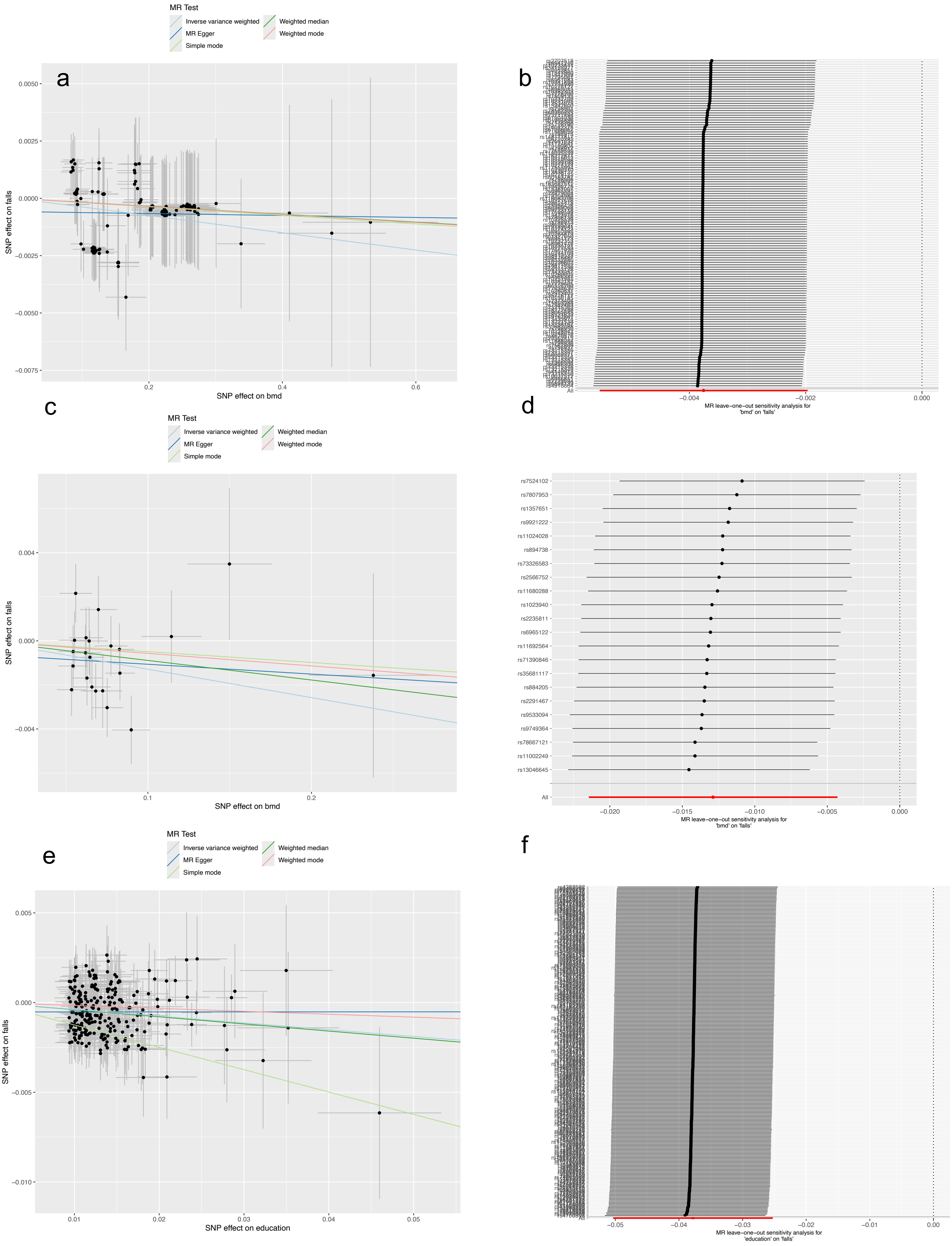

Supplemental Figure 1: Sensitivity analysis results featuring leave-one-out plots and funnel plots. (a) Leave-one-out plot showing the causal impact of FA-BMD on falls after excluding each SNP in turn. (b) Funnel plot illustrating the overall symmetry of causal effects across all instrumental variables for FA-BMD. (c) Leave-one-out plot for FN-BMD, presenting the sensitivity analysis results of the relationship between FN-BMD and falls. (d) Funnel plot for FN-BMD, displaying the symmetry of causal effects across all instrumental variables. (e) Leave-one-out plot for years of education, detailing the sensitivity analysis results of the relationship between education and falls. (f) Funnel plot for years of education, showing the overall symmetry of causal effects across all instrumental variables for education.
